# Prediction of low birth weight with hypoglycemia in glucose tolerance test

**DOI:** 10.11606/s1518-8787.2021055002543

**Published:** 2021-05-06

**Authors:** Flavio Hernández-Castro, Anaís Berlanga-Garza, Mayela Diamantina Cruz-Gutiérrez, Juan Antonio Soria-López, Gabriel Edgar Villagómez-Martínez, Iván Vladimir Dávila-Escamilla

**Affiliations:** I Universidad Autónoma de Nuevo León Hospital Universitario Dr. José Eleuterio González Departamento de Obstetricia y Medicina Materno Fetal Nuevo León México Universidad Autónoma de Nuevo León (UANL). Hospital Universitario Dr. José Eleuterio González. Departamento de Obstetricia y Medicina Materno Fetal. Monterrey, Nuevo León, México

**Keywords:** Hypoglycemia, Glucose Tolerance Test, Infant, Low Birth Weight, Longitudinal Studies

## Abstract

**OBJECTIVE:**

Determine the value of the combination of fasting glucose less than the 10th percentile (FG < p10) during 75 gram oral glucose tolerance test (75g OGTT) with maternal characteristics to predict low birth weight (LBW) established by Intergrowth-21st tables.

**METHODS:**

Prospective cohort study of pregnant women who was underwent 75g OGTT between 24 and 28.6 weeks. The 10th percentile fasting glucose of the population was determined at 65 mg/dL and women with risk factors that could modify fetal weight, including those related to intrauterine growth restriction, were excluded. Two groups were formed: group FG < p10 and group with normal fasting glucose. The main finding was the diagnosis of LBW. The association between FG < p10, maternal characteristics and LBW was established by multivariate logistic regression. The predictive performance of the models constructed was evaluated by receiver operating characteristic (ROC) curve and area under the curve (AUC) analysis.

**RESULTS:**

349 women were eligible for study, of whom 66 (18.91%) had FG < p10; neonates in this group had lower birth weights (2947.28 g and 3138.26 g, p = 0.001), higher frequencies of LBW (25% and 6.81%, p < 0.001) and of weights < 2500 g in term births (8.6% and 2.3%, p = 0.034). The basal prediction model consisted of nulliparity by achieving an AUC of 60%, while the addition of FG < p10 resulted in the significant improvement of the previous model (AUC 72%, DeLong: p = 0.005).

**CONCLUSIONS:**

In pregnant women without factors that could modify fetal weight, the predictive model created by combining FG < p10 during 75g OGTT with nulliparity was significantly associated with increased risk of LBW.

**REGISTRATION:**

ClinicalTrials.gov: NCT04144595.

## INTRODUCTION

Low birth weight (LBW) of neonates is a global public health problem. In 2015 according to estimates by the World Health Organization (WHO), 1 in 7 live newborns (20.5 million) in the world had LBW ^1^. In addition, more than 80% of neonatal deaths are related to LBW and, of these, two thirds occur in preterm births ^1^^,^^2^.

Low fasting serum glucose (FSG) levels during pregnancy have been associated with LBW since the 1970s. Multiple FG values (< 61 mg/dL to < 87 mg/dL) have been used as a cut-off point to establish this association^3^^–^^6^; no universally accepted number is currently available.

Hypoglycemia in pregnancy is the result of a relative hyperinsulinemic state that may be due to increased levels of insulin or its receptors but also to a decrease in one or more of the diabetogenic hormones such as placental lactogen^7^^,^^8^. Glucose is the main energy substrate for the fetus. Fetal serum glucose concentration changes as a function of maternal concentration and gestational age^6^. Under normal conditions there is no fetal gluconeogenesis, so it depends on the facilitated diffusion of glucose from the mother through placental transporter proteins of the GLUT family^9^. A relative maternal hypoglycemia would lead to acute or chronic decrease in fetal glucose and insulin and, considering that insulin is a hormone involved in fetal growth, would hypothetically predispose to intrauterine growth restriction (IUGR) and LBW^10^.

Abnormal maternal glucose metabolism could lead to an adverse intrauterine environment and increase obstetric complications. Thus, the American Diabetes Association (ADA) recommends the 75 gram oral glucose tolerance test (75g OGTT) for the diagnosis of gestational diabetes (GD) between 24 and 28 weeks of pregnancy. Although in this test a FG value ≥ 92 mg/dL is considered abnormal^11^, the lower limit is not defined.

Although previous research suggested that hypoglycemia during pregnancy is associated with LBW and poor neurodevelopment^12^, landmark GD studies such as the Hyperglycemia and Adverse Pregnancy Outcome (HAPO)^13^ and the Australian Carbohydrate Intolerance Study in Pregnant Women (ACHOIS)^14^ did not address the risks of maternal hypoglycemia and its effect on perinatal outcomes.

On the other hand, because of the important influence that birth weight has on the short and long term prognosis of individuals, the international project Intergrowth-21st (IG21) proposed a global standard both to define and classify birth weights and to facilitate clinical decision making based on these weights^15^.

Until now, there is no information in Mexico about the association between low FG with LBW, but any cut-off point in FG was proposed to establish it. The aim of this study was to determine the value of combining low FG during 75g OGTT with maternal characteristics to predict LBW defined by IG21 tables.

## METHODS

### Study Design and Selection of Patients

Prospective cohort study conducted at the Department of Obstetrics of the Hospital Universitario Dr. José Eleuterio González of the Universidad Autónoma de Nuevo León in Monterrey, Mexico. Received approval from the university ethics committee (GI19-00006) and registration in ClinicalTrials.gov:NCT04144595.

In January 2019, in a pilot trial conducted under ethical standards and including 200 pregnant women, the 10th percentile of FG during 75g OGTT of the population to be studied (65 mg/dL) was determined; for the purposes of the research, for values lower than this, GA was considered low. Patients were recruited between March and November 2019.

The sample size was calculated based on the difference in proportions of newborns with LBW reported in women with low glucose during the oral glucose tolerance test^16^, with a significance level of 0.05, power of 80% and, considering a 5:1 ratio between the control and study groups, it was estimated necessary to recruit at least 317 patients.

All pregnant women who underwent 75g OGTT between 24 and 28.6 weeks of gestation were invited to participate. Before being recruited, the objective of the research was explained, the confidential nature of the information and informed consent was requested. Inclusion criteria were: pregnancy with a single fetus and reliable amenorrhea or first trimester ultrasound to establish gestational age. Serum glycemia values were recorded at 0.60 and 120 minutes. With those results, participants with GD diagnosed as established by the ADA with at least one of the following values were excluded: FG ≥ 92 mg/dL, 1 hour ≥ 180 mg/dL, 2 hours ≥ 153 mg/dL^11^. Those with comorbidities that could modify fetal growth were also excluded: pregestational diabetes, systemic lupus erythematosus, antiphospholipid antibody syndrome and other thrombophilias, heart disease, chronic pulmonary or renal disease, and thyroid disease. In addition, any hypertensive disease in pregnancy or derived complications (chronic arterial hypertension, gestational hypertension, pre-eclampsia, eclampsia). Patients with exposure to substances that could affect fetal weight were excluded: teratogens (cyclophosphamide, valproic acid, antithrombotic drugs), tobacco, alcohol, cocaine or coffee consumption of more than 1 cup/day^17^^,^^18^. Finally, women with a history of intrauterine growth restriction (IUGR) and/or preeclampsia in previous pregnancies, high risk of IUGR and/or preeclampsia in the current pregnancy estimated at first trimester screening, mean uterine artery greater than the 95th percentile for gestational age at 24 weeks, fetal structural defects or markers of aneuploidy on first or second trimester ultrasonography were also excluded^19^.

Patients who developed intra-amniotic infections (cytomegalovirus, rubella, toxoplasmosis, herpes or syphilis), IUGR or preeclampsia during gestation^17^ were eliminated from the study, as well as those whose newborns had structural defects not diagnosed prenatally, those who had incomplete clinical records or who ended their pregnancy in another institution.

### Definition of Variables

The main finding was the diagnosis of LBW, defined for the purposes of the study as birth weight less than the 10th percentile for gestational age (BW< p10), the secondary findings were the diagnoses of birth weight less than the 3rd percentile (BW < p3) and < 2500 gram. The birth weight percentiles were established using the tables for gestational age and gender defined in IG21^15^.

Maternal characteristics collected were: age, body mass index (BMI) during 75g OGTT dichotomized as established by the American College of Obstetricians and Gynecologists into underweight (BMI < 18. 5 kg/m^2^) and obesity (BMI ≥ 30 kg/m^2^)^20^, gestational weight gain dichotomized as described by the Institute of Medicine into low and excessive according to weight gain in kg from BMI at the start of pregnancy^21^, parity, gestational age when 75g OGTT was performed, diagnosis of anemia (Hb < 11 g/dL)^22^ during the third trimester of pregnancy, and FG.

### Study Groups

Two groups were formed according to FG during 75g OGTT: study group (FG < 10th percentile) and control group (FG ≥ 10th percentile and < 92 mg/dL). No clinical decision was made based on the finding of FG < 10th percentile, as this result was blinded for both participants and obstetrics and pediatrics personnel. During prenatal care, the diagnosis and follow-up of fetuses weighing less than the 10th percentile for gestational age (FP < p10) in either group was according to intrahospital guidelines based on international protocols and not on the result of the FG^23^.

The neonatal data analyzed were: gestational age, gender, Apgar score and birth weight. The latter was classified according to the percentile defined in IG21.^15^.

### Statistical Analysis

The distribution of quantitative variables was established using the Kolmogorov-Smirnov test. Those with parametric distribution were expressed as mean (95% confidence interval) and compared with Student’s t test. Nonparametric variables were described as median (interquartile range) and contrasted with the Mann-Whitney U test. Categorical variables were compared with Pearson’s X^2^ test or Fisher’s exact test.

The association between FG < 10th percentile with BW < p3, BW < p10, weight < 2500 gram and maternal characteristics (age ≥ 35 years, nulliparity, low weight gain during pregnancy, low BMI or obesity at the time of 75g OGTT and anemia)^22^^,^^24^ was evaluated by multivariate logistic regression analysis.

The basal model included risk factors for LBW^23^^,^^24^. Maximum models were generated involving all the independent variables, and, from these, final models including significant and nonsignificant variables whose exclusion modified the coefficients of other variables by more than 10%. The models were compared by establishing the improvement in their Nagelkerke R^2^ statistic as a measure of goodness-of-fit using X^2^ Wald, their predictive capacity was determined with receiver operating characteristic (ROC) and area under the curve (AUC) analysis. SPSS version 22.0 (SPSS Inc, Illinois, USA) and MedCalc Statistical Software version 19.1.5 (MedCalc Software bv, Ostend, Belgium; https://www.medcalc.org; 2020) were used for statistical analysis. All statistical tests were considered significant at p values < 0.05.

## RESULTS

**Figure 1:**
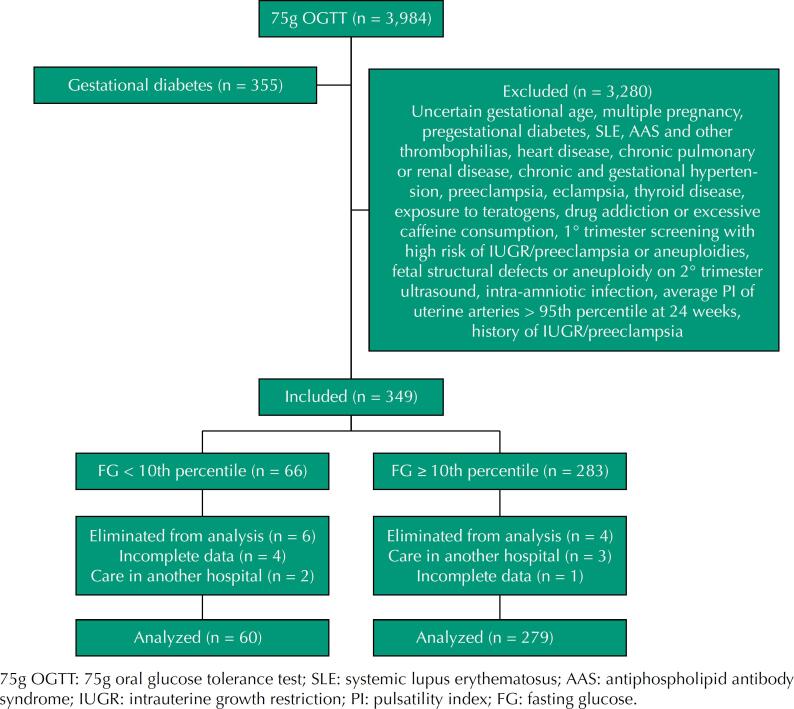
Flow diagram of the groups studied.

During the recruitment period, the 75g OGTT results of 3984 women were reviewed, of whom 355 (8.91%) were excluded for a diagnosis of GD and 3280 for having any risk factor that could decrease fetal weight. A total of 349 were eligible for study and, of these, 10 (2.87%) were eliminated (Figure 1). Of the patients included, 66 (18.91%) had FG < 65 mg/dL, none had symptoms of hypoglycemia.

### Maternal Characteristics

The characteristics of women in the FG < 10th percentile and control groups were similar except for FG which was significantly lower in the study group (Table 1).

**Table 1:** Maternal characteristics according to fasting glucose in 75g OGTT.

Characteristics	Fasting glucose		p
	< percentile 10 (n = 60)	≥ percentile 10 (n = 279)	
Age (years)	26 (20–31)	23 (20–28)	0.127^a^
< 19	8 (13.33)	52 (18.63)	0.329^b^
≥ 35	5 (8.33)	22 (7.89)	0.907^b^
BMI during 75g OGTT (Kg/m2)	26.9 (23.6–29.8)	26.1(22.5–29.8)	0.394^a^
Underweight	0	4 (1.43)	1^b^
Obese	15 (25)	66 (23.66)	0.825^b^
Gestational weight gain (kg)	10 (8–12)	9 (7–12)	0.367^a^
Low weight gain	18 (30)	114 (40.86)	0.118^b^
Excessive weight gain	17 (28.33)	68 (24.37)	0.521^b^
Parity	2 (1–3)	1(1–3)	0.824^a^
Nulliparity	28 (46.67)	141(50.53)	0.587^b^
Gestational age during 75g OGTT	26.4 (25.5–27.6)	26.4 (25.2–27.6)	0.663^a^
Anemia (< 11 g/dL)	16 (26.67)	79 (28.31)	0.796^b^
Fasting glucose (mg/dL)	60 (57–61.5)	80 (73–86)	< 0.001^a^

75g OGTT: 75g oral glucose tolerance test; BMI: body mass index.

a Variables with nonparametric distribution presented as median (interquartile range) and compared with the Mann-Whitney U test.

b Frequencies presented as n (%) and compared with Pearson’s X^2^ or Fisher’s exact test.

### Neonatal Results

Neonates in the FG < 10th percentile group were characterized by lower birth weights and IG21 percentiles, as well as having higher frequencies of BW < p3, BW < p10 and weights < 2500 gram at term births (8.6% and 2.3%, p = 0.034). There were no differences between groups with respect to gestational age at birth, neonatal gender, Apgar score at 5 minutes or weight < 2500 gram when including preterm births (Table 2).

**Table 2:** Neonatal characteristics according to fasting glucose in 75g OGTT.

Characteristics	Fasting glucose		p
	< percentile 10 (n = 60)	≥ percentile 10 (n = 279)	
Age at birth (weeks)	39 (38.4–39.6)	39(38.1–40)	0.356^a^
< 34	1 (1.67)	2 (0.72)	0.443^b^
< 37	2 (3.33)	22 (7.88)	0.275^b^
> 41	2 (3.33)	5 (1.79)	0.359^b^
Birth weight (gram)	2947.28 (2833.82–3060.75)	3138.26 (3091.9–3184.7)	0.001^c^
< 2,500	6 (10)	13 (4.66)	0.103^b^
> 4,000	0	3 (1.07)	1^b^
Percentile of birth weight IG21	29.32 (9.93–54.61)	49.24 (28.5–70.1)	< 0.001^a^
< 3	5 (8.33)	5 (1.79)	0.006^b^
< 10	15 (25)	19 (6.81)	< 0.001^b^
> 90	2 (3.33)	15 (5.38)	0.511^b^
Gender of neonate			
Female	24 (40)	145 (52)	0.092^b^
Male	36 (60)	134 (48)	0.092^b^
Apgar < 7 at 5 minutes	5(8.33)	16 (5.7)	0.449^b^

1 75g OGTT: 75g oral glucose tolerance test; IG21: Intergrowth-21st Project.

a Variables with nonparametric distribution presented as median (interquartile range) and compared with the Mann-Whitney U test.

b Frequencies presented as n (%) and compared with Pearson’s X^2^ or Fisher’s exact test.

c Variables with parametric distribution presented as mean (95%CI) and compared with Student’s t test.

The correlation between FG and birth weight was significant in the FG group < p10 (Spearman correlation coefficient = 0.274, 95%CI 0.22-0.49, p = 0.034).

### Low Birth Weight Prediction

Multivariate logistic regression analysis was used to establish the association between FG < 10th percentile with BW < p3, BW < p10, weight < 2500 gram and maternal characteristics while controlling for potentially confounding variables such as: maternal age, BMI during 75g OGTT, gestational weight gain, parity, anemia and neonatal gender.

**Figure 2:**
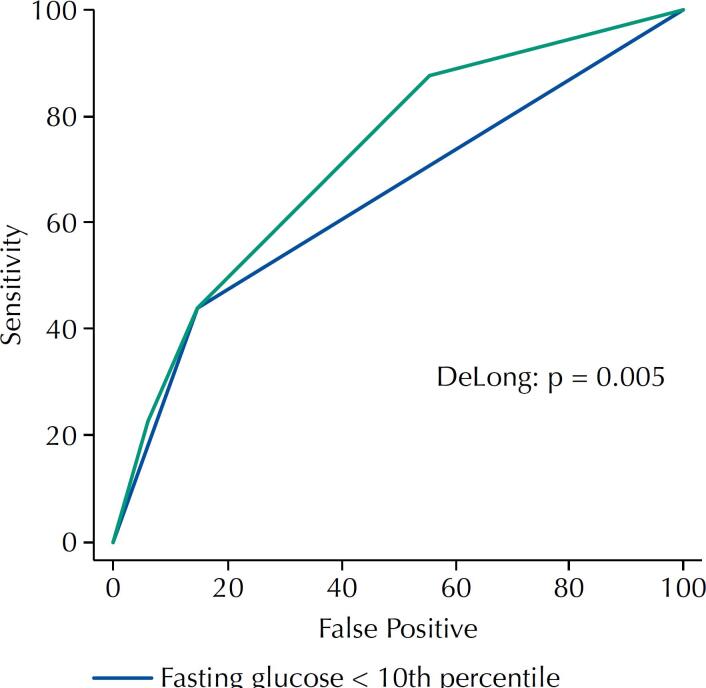
Performance of predictive models of birth weight < 10th percentile according to Intergrowth-21st based on 75g OGTT fasting glucose.

Of the maternal characteristics, the only one significantly associated with BW < p10 was nulliparity with an OR 2.28, 95%CI 1.07–4.83, p = 0.032. This variable was used to construct the basal model to predict LBW with Nagelkerke R^2^ of 2.9% and AUC of 0.60, 95%CI 0.55–0.65. FG < 10th percentile was independently associated with increased risks of BW < p3 (OR 4.98, 95%CI 1.4–17.8, p = 0.018), BW < p10 (OR 4.56, 95%CI 2.16–9.63, p < 0.001) and weights < 2500 gram (OR 3.95, 95%CI 1.16–13.41, p = 0.04), but the latter only in term pregnancies. The prediction basal model in FG < 10th percentile had a Nagelkerke’s R^2^ of 8.8%. Adding FG < 10th percentile to the basal model showed a Nagelkerke’s R^2^ of 12.2%, AUC 0.72, 95%CI 0.67–0.77 significantly improving the previous model (DeLong: p = 0.005). Table 3 details the characteristics of the logistic regression models created and the comparisons between them. Figure 2 shows the AUC of the models created.

**Table 3:** Models constructed for the prediction of low birth weight established by IG21.

Model	OR	95%CI	p	AUC	95%CI	Sen (%)	Spe (%)	PPV (%)	NPV (%)
Maternal characteristics: Nulliparity	2.28	1.07–4.83	0.032	0.60	0.55–0.65	67.61	52.15	13.63	93.52
FG < 10th percentile	4.56	2.16–9.63	< 0.001	0.65	0.59–0.70	44.13	85.29	25	93.21
Nulliparity + FG < percentile 10	2.52 4.91	1.16–5.49 2.29–10.52	0.022 < 0.001	0.72	0.67–0.77^a^	23.53	93.44	28.67	91.61

IG21: Intergrowth-21st; AUC: area under the curve; FG: Fasting glucose.

a Comparison of AUC with previous model, DeLong: p = 0.005.

## DISCUSSION

These results present that, when excluding risk factors and controlling for confounding variables with potential negative effect on fetal weight, FG < 10th percentile during 75g OGTT independently predicts LBW. The only maternal characteristic, besides FG < 10th percentile, with influence on birth weight was nulliparity, and the result of combining both variables was to increase their ability to predict LBW.

Although multiple risk factors have been cited as possible causes of IUGR, many of these fetuses remain undetected by ultrasonography until birth and a large number of metabolic causes was not established^10^^,^^23^.

Some fetuses weighing less than the 10th percentile for gestational age of unexplained cause have hypoglycemia and in these pregnancies the mothers have 22% higher insulin sensitivity with lower plasma glucose, insulin and placental lactogen levels after glucose tolerance testing compared to those with normal weight fetuses^7^^,^^25^. It is likely that the main cause of hypoglycemia in these fetuses is a decreased glucose intake from the mother and/or an increased placental glucose consumption but not a decrease in the placental density of glucose transporters or a decrease in their transport capacity through the syncytiotrophoblast^25^^,^^26^.

Identification of the cause of LBW is essential to establish prevention and treatment strategies. It is not always possible to attribute it to well-known risk factors such as aneuploidies, structural defects, placental insufficiency or infections^27^. The above was verified in the present study by excluding women with such characteristics and with the observation that only 8.8% of the variance of BW < p10 was explained by FG <percentile 10, therefore, a causal relationship could not be established.

The data presented are similar to previous research that found a relationship between low serum glucose levels during GD screening with LBW^3^^–^^6^. Studies such as the one by Naik et al. used lower serum glucose cut points (≤ 50 mg/dL) to define hypoglycemia and have found no association with low birth weight^28^, but unlike the present investigation included pregnancies exceeding 28 weeks with a diagnosis of GD on insulin therapy, did not differentiate other risk factors, and used continuous glucose monitoring devices that recorded > 90% of hypoglycemia episodes between 23:00 and 06:00 hours unrelated to 75g OGTT. The use of that cutoff point in our population would not have clinical applicability, as the incidence of FG ≤50 mg/dL would be 0.86% with AUC to predict LBW of 0.51, 95%CI 0.46-0.57.

Similar to other reports, we also found no association with preterm birth, low Apgar, or maternal morbidity ^5^^,^^10^^,^^27^. On the other hand, these results contrast with those of Calfee et al.^29^, who found no difference in the incidence of IUGR between women with low glucose levels and those with normal glucose. In the study, the group of women with low glucose were younger and underweight, and included a greater number of adolescents and nulliparous women than the control group. These differences compared to our research could be explained by considering the following: there was no ethnic diversity in the population studied, the basal characteristics of the groups studied were homogeneous, and although we sought to control for BMI categories as a risk factor for LBW, 59% of the patients included were overweight or obese, which may have decreased maternal insulin sensitivity and modified fetal growth.

In this analysis, maternal age was not a factor associated with LBW, contrary to the study by Pugh et al. who described that women with hypoglycemia were younger and with lower pregestational BMI^30^. This difference may be based on the fact that these women also had pre-pregnancy diseases that could modify birth weight, an item that was excluded in the present study in order to avoid confounding bias.

Our research has several assets, so far we know that it is the first national study that reported the 10th percentile of FG during 75g OGTT in pregnant women, as well as the association between FG below that cutoff point with neonatal weight classified by IG21. Furthermore, it was the prospective design in which unlike previous studies (mostly retrospective)^3^^,^^5^^,^^6^^,^^16^^,^^27^, most risk factors related to fetuses small for gestational age and IUGR were excluded and strictly controlled.

Some limitations should be considered: first, a potential selection bias because the study was performed in only one third level referral center with a high prevalence of GD and risk factors that could affect fetal growth. Second, although we tried to exclude and control characteristics considered risk factors for IUGR and LBW, it would be possible to find unknown maternal, placental or fetal factors with the potential to modify birth weight. Third, this study included only Mexican women; therefore, external validity could be limited by the homogeneity of the cohort.

Finally, potential confounding variables that could modify birth weight such as socioeconomic status, pregestational BMI, caloric intake during gestation and serum lipid levels were not obtained.

## CONCLUSION

In pregnant women without risk factors that could modify fetal weight, the predictive model created by combining FG < p10 during 75g OGTT with nulliparity was significantly associated with increased risk of LBW. Although the discriminatory capacity of the proposed model was moderate, it could represent a useful clinical tool to identify women at high risk of having a low birth weight neonate, in addition to providing an opportunity for possible prophylactic actions, as well as to design predictive models involving fetal ultrasonographic parameters in order to decrease the rate of infant morbidity and mortality related to LBW.
