# Supplementary material for: Prediction of low birth weight with hypoglycemia in glucose tolerance test
Source: Rev Saude Publica. 2021 May 6;55:30. doi: 10.11606/s1518-8787.2021055002543 (PMC8102028; doi:10.11606/s1518-8787.2021055002543)
Supplement: Translation

# Translation: Predicción de bajo peso al nacer con hipoglucemia en la prueba de tolerancia a la glucosa

**Authors:** Flavio Hernández-Castro, Anaís Berlanga-Garza, Mayela Diamantina Cruz-Gutiérrez, Juan Antonio Soria-López, Gabriel Edgar Villagómez-Martínez, Iván Vladimir Dávila-Escamilla

**Affiliations:** I Nuevo León México Universidad Autónoma de Nuevo León (UANL). Hospital Universitario Dr. José Eleuterio González. Departamento de Obstetricia y Medicina Materno Fetal. Monterrey, Nuevo León, México

**Keywords:** Hipoglucemia, Prueba de Tolerancia a la Glucosa, Recién Nacido de Bajo Peso, Estudios Longitudinales

## Abstract

**OBJETIVO:**

Determinar el valor de la combinación de la glucosa en ayunas menor que el percentil 10 (GA < p10) durante la prueba de tolerancia oral a la glucosa con 75 gramos (PTG-75g) con características maternas para predecir bajo peso al nacer (BPN) establecido mediante tablas de Intergrowth-21st.

**MÉTODOS:**

Estudio de cohorte prospectivo de mujeres embarazadas que se realizaron PTG-75g entre las 24 y 28.6 semanas. Se determinó el percentil 10 de glucosa en ayunas de la población en 65 mg/dL y fueron excluidas aquellas mujeres con factores de riesgo que pudieran modificar el peso fetal incluyendo los relacionados con la restricción del crecimiento intrauterino. Se formaron dos grupos: grupo GA < p10 y grupo con glucosa en ayunas normal. El hallazgo principal fue el diagnóstico de BPN. La asociación entre GA < p10, características maternas y BPN se estableció mediante regresión logística multivariante. El desempeño predictivo de los modelos construidos fue evaluado por el análisis de la curva característica operativa del receptor (ROC) y del área bajo la curva (ABC).

**RESULTADOS:**

Fueron elegibles para estudio 349 mujeres, de las cuales 66 (18,91%) tuvieron GA < p10; los neonatos de este grupo tuvieron pesos al nacer más bajos (2947.28 g y 3138.26 g, p = 0,001), frecuencias más altas de BPN (25% y 6,81%, p < 0,001) y de pesos < 2500 g en nacimientos de término (8,6% y 2,3%, p = 0,034). El modelo basal de predicción consistió en nuliparidad al lograr un ABC del 60%, mientras que al añadir la GA < p10 se obtuvo la mejora significativa del modelo previo (ABC 72%, DeLong: p = 0,005).

**CONCLUSIONES:**

En mujeres embarazadas sin factores que pudieran modificar el peso fetal, el modelo predictivo creado combinando GA < p10 durante la PTG-75g con nuliparidad estuvo asociado significativamente con riesgo incrementado de BPN.

**REGISTRO:**

ClinicalTrials.gov: NCT04144595.

## INTRODUCCIÓN

El bajo peso al nacer (BPN) de neonatos es un problema mundial de salud pública. En 2015 según estimaciones de la Organización Mundial de la Salud (OMS), 1 de cada 7 recién nacidos vivos (20.5 millones) en el mundo tuvo BPN^1^. Además, más de 80% de las muertes neonatales se relacionan con BPN y, de estas, dos terceras partes ocurren en nacimientos pretérmino^1^^,^^2^.

Los niveles bajos de glucosa sérica en ayunas (GA) durante el embarazo han sido relacionados con BPN desde la década de 1970. Múltiples valores de GA (< 61 mg/dL a < 87 mg/dL) han sido utilizados como punto de corte para establecer esta asociación^3^^–^^6^; a la fecha no existe una cifra universalmente aceptada.

La hipoglucemia en el embarazo es el resultado de un estado hiperinsulinémico relativo que puede deberse al incremento en los niveles de insulina o sus receptores, pero también a la disminución de una o más de las hormonas diabetogénicas como el lactógeno placentario^7^^,^^8^. La glucosa es el principal sustrato energético para el feto. La concentración fetal de glucosa sérica cambia en función de la concentración materna y la edad gestacional^6^. En condiciones normales no hay gluconeogénesis fetal por lo que depende de la difusión facilitada de la glucosa proveniente de la madre a través de proteínas transportadoras placentarias de la familia GLUT^9^. Es decir, una relativa hipoglucemia materna conduciría a disminución aguda o crónica de glucosa e insulina fetales y, considerando que la insulina es una hormona involucrada en el crecimiento fetal, hipotéticamente predispondría a la restricción del crecimiento intrauterino (RCIU) y al BPN^10^.

Puesto que el metabolismo materno anormal de la glucosa podría originar un ambiente intrauterino adverso e incrementar las complicaciones obstétricas, la American Diabetes Association (ADA) recomienda la prueba de tolerancia oral a la glucosa con 75 gramos (PTG-75g) para el diagnóstico de diabetes gestacional (DG) entre las 24 y 28 semanas de embarazo. Aunque en esta prueba el valor de la GA ≥ 92 mg/dL es considerado anormal^11^, el límite inferior no ha sido definido.

A pesar de que investigaciones previas han sugerido que la hipoglucemia durante el embarazo se asocia con BPN y un pobre neurodesarrollo^12^, estudios emblemáticos sobre DG como el *Hyperglycemia and Adverse Pregnancy Outcome* (HAPO)^13^ y el *Australian Carbohydrate Intolerance Study in Pregnant Women* (ACHOIS)^14^ no abordaron los riesgos de la hipoglucemia materna y su efecto sobre los resultados perinatales.

Por otra parte, debido a la importante influencia que tiene el peso al nacer sobre el pronóstico a corto y largo plazo de los individuos, el proyecto internacional Intergrowth-21st (IG21) propuso un estándar mundial tanto para definir y clasificar los pesos al nacimiento como para facilitar la toma de decisiones clínicas basadas en ellos^15^.

Hasta el momento, no existe información en México acerca de la asociación entre GA baja con BPN y mucho menos se ha propuesto algún punto de corte en la GA para establecerla. El objetivo de este estudio fue determinar el valor de la combinación de la GA baja durante la PTG-75g con características maternas para predecir BPN definido mediante tablas de IG21.

## MÉTODOS

### Diseño del Estudio y Selección de Pacientes

Estudio de cohorte prospectivo realizado en el Departamento de Obstetricia del Hospital Universitario Dr. José Eleuterio González de la Universidad Autónoma de Nuevo León en Monterrey, México. Recibió aprobación del comité de ética universitario (GI19-00006) y registro en ClinicalTrials.gov:NCT04144595.

En enero de 2019, en una prueba piloto que se condujo bajo los estándares éticos y que incluyó a 200 mujeres embarazadas, se determinó el percentil 10 de la GA durante la PTG-75g de la población que estudiar (65 mg/dL); según los propósitos de la investigación para los valores inferiores a ese se consideraron GA baja. Se reclutaron a las pacientes entre marzo y noviembre de 2019.

El tamaño de la muestra fue calculado con base en la diferencia de proporciones de recién nacidos con BPN reportada en mujeres con glucosa baja durante la prueba de tolerancia oral a la glucosa^16^, con nivel de significancia de 0,05, poder del 80% y, considerando una relación 5:1 entre los grupos control y de estudio, se estimó necesario reclutar, como mínimo, a 317 pacientes.

Se invitó a participar a todas las mujeres embarazadas que se realizaron PTG-75g entre las 24 y 28.6 semanas de gestación. Antes de ser reclutadas, se explicó el objetivo de la investigación, el carácter confidencial de la información y se solicitó el consentimiento informado. Se consideraron criterios de inclusión: cursar embarazo con feto único y tener amenorrea confiable o ultrasonido de primer trimestre para establecer edad gestacional. Se registraron los valores de glucemia sérica a los 0, 60 y 120 minutos. Con esos resultados se excluyeron las participantes con DG diagnosticada según lo establecido por la ADA con al menos uno de los siguientes valores: GA ≥ 92 mg/dL, 1 hora ≥ 180 mg/dL, 2 horas ≥ 153 mg/dL^11^. Fueron excluidas también aquellas con comorbilidades que pudieran modificar el crecimiento fetal: diabetes pregestacional, lupus eritematoso sistémico, síndrome de anticuerpo antifosfolípido y otras trombofilias, cardiopatías, enfermedades pulmonares o renales crónicas y enfermedades tiroideas. Además, cualquier enfermedad hipertensiva en el embarazo o complicaciones derivadas (hipertensión arterial crónica, hipertensión gestacional, preeclampsia, eclampsia). Se excluyeron a pacientes con exposición a sustancias que pudieran afectar el peso fetal: teratógenos (ciclofosfamida, ácido valpróico, fármacos antitrombóticos), tabaco, alcohol, cocaína o consumo de café superior a 1 taza/día^17^^,^^18^. Finalmente, las mujeres con antecedente de restricción del crecimiento intrauterino (RCIU) y/o preeclampsia en embarazos previos, riesgo alto de RCIU y/o preeclampsia en el embarazo actual estimado en tamizaje de primer trimestre, índice de pulsatilidad promedio de arterias uterinas mayor que el percentil 95 para la edad gestacional a las 24 semanas, defectos estructurales fetales o marcadores de aneuploidías en ultrasonografías de primer o segundo trimestres también fueron excluidas^19^.

Se eliminaron del estudio a las pacientes que desarrollaron infecciones intraamnióticas (citomegalovirus, rubeola, toxoplasmosis, herpes o sífilis), RCIU o preeclampsia durante la gestación^17^, así como aquellas cuyos recién nacidos tuvieron defectos estructurales no diagnosticados prenatalmente, las que tuvieron expediente clínico incompleto o terminaron su embarazo en otra institución.

### Definición de Variables

El hallazgo principal fue el diagnóstico de BPN, definido para propósitos del estudio como peso al nacimiento menor que el percentil 10 para la edad gestacional (PN < p10), los secundarios fueron los diagnósticos de peso al nacer menor que el percentil 3 (PN < p3) y < 2500 gramos. Los percentiles del peso al nacimiento fueron establecidos mediante las tablas para edad gestacional y género asignadas en IG21^15^.

Las características maternas recolectadas fueron: edad, índice de masa corporal (IMC) durante la PTG-75g dicotomizada según lo establecido por el American College of Obstetricians and Gynecologists en bajo peso (IMC < 18.5 kg/m^2^) y obesidad (IMC ≥ 30 kg/m^2^)^20^, ganancia de peso gestacional dicotomizada de acuerdo a lo descrito por el Institute of Medicine en baja y excesiva según el aumento de peso en kg a partir del IMC al inicio del embarazo^21^, paridad, edad gestacional en que se realizó la PTG-75g, diagnóstico de anemia (Hb < 11 g/dL)^22^ durante el tercer trimestre del embarazo y GA.

### Grupos de Estudio

Se formaron dos grupos según la GA durante la PTG-75g: grupo de estudio (GA < percentil 10) y grupo control (GA ≥ percentil 10 y < 92 mg/dL). No se tomó ninguna decisión clínica con base en el hallazgo de GA <percentil 10, pues este resultado fue cegado tanto para las participantes como para el personal de obstetricia y pediatría. Durante el control prenatal, el diagnóstico y seguimiento de los fetos con peso menor que el percentil 10 para la edad gestacional (PF < p10) de cualquier de los dos grupos fue acorde a lineamientos intrahospitalarios basados en protocolos internacionales y no al resultado de la GA^23^.

Los datos neonatales analizados fueron: edad gestacional, género, puntuación en escala Apgar y peso al nacer. Este último lo clasificaron según el percentil asignado en IG21^15^.

### Análisis Estadístico

La distribución de las variables cuantitativas fue establecida mediante la prueba de Kolmogorov-Smirnov. Las que tuvieron distribución paramétrica fueron expresadas como media (intervalo de confianza del 95%) y comparadas con prueba t de Student. Las no paramétricas fueron descritas como mediana (intervalo intercuartil) y contrastadas con prueba U de Mann-Whitney. Las variables categóricas fueron comparadas con pruebas *X*^2^ de Pearson o exacta de Fisher.

La asociación entre GA <percentil 10 con PN < p3, PN < p10, peso < 2500 gramos y características maternas (edad ≥ 35 años, nuliparidad, baja ganancia de peso durante el embarazo, IMC bajo u obesidad al momento de la PTG-75g y anemia)^22^^,^^24^ fue evaluada mediante análisis de regresión logística multivariante.

El modelo basal incluyó factores de riesgo para BPN^23^^,^^24^. Se generaron modelos máximos que abarcaron todas las variables independientes y, a partir de ellos, modelos finales que incluyeron variables significativas y no significativas cuya exclusión modificaba los coeficientes de otras variables más del 10%. Se compararon los modelos estableciendo la mejora en su estadístico R^2^ de Nagelkerke como medida de bondad de ajuste usando *X*^2^ Wald, su capacidad predictiva fue determinada con análisis de la curva característica operativa del receptor (ROC) y área bajo la curva (ABC). Para el análisis estadístico se utilizaron los programas SPSS versión 22.0 (SPSS Inc, Illinois, EE.UU.) y MedCalc *Statistical Software* versión 19.1.5 (MedCalc *Software* bv, Ostend, Bélgica; https://www.medcalc.org; 2020). Todas las pruebas estadísticas se consideraron significativas con valores de p < 0,05.

## RESULTADOS

Durante el periodo de reclutamiento, se revisaron los resultados de la PTG-75g de 3984 mujeres, de las cuales fueron excluidas 355 (8,91%) por diagnóstico de DG y 3280 por tener algún factor de riesgo que pudiera disminuir el peso fetal. Un total de 349 fueron elegibles para estudio y, de estas, 10 (2,87%) fueron eliminadas (Figura 1). De las pacientes incluidas, 66 (18,91%) tuvieron GA < 65 mg/dL, ninguna tuvo sintomatología de hipoglucemia.

**Figura 1:** Diagrama de flujo de los grupos estudiados.

### Características Maternas

Las características de las mujeres de los grupos GA < percentil 10 y control fueron similares excepto por la GA que fue significativamente más baja en el grupo de estudio (Tabla 1).

**Tabla 1:** Características maternas según la glucemia en ayunas en la PTG-75g.

Características	Glucemia en ayunas		p
	< percentil 10 (n = 60)	≥ percentil 10 (n = 279)	
Edad (años)	26 (20–31)	23 (20–28)	0,127^a^
< 19	8 (13.33)	52 (18.63)	0,329^b^
≥ 35	5 (8.33)	22 (7.89)	0,907^b^
IMC durante PTG-75g (Kg/m^2^)	26.9 (23.6–29.8)	26.1(22.5–29.8)	0,394^a^
Bajo peso	0	4 (1.43)	1^b^
Obesidad	15 (25)	66 (23.66)	0,825^b^
Ganancia de peso gestacional (kg)	10 (8–12)	9 (7–12)	0,367^a^
Baja ganancia de peso	18 (30)	114 (40.86)	0,118^b^
Excesiva ganancia de peso	17 (28.33)	68 (24.37)	0,521^b^
Paridad	2 (1–3)	1(1–3)	0,824^a^
Nuliparidad	28 (46.67)	141(50.53)	0,587^b^
Edad gestacional durante PTG-75g	26.4 (25.5–27.6)	26.4 (25.2–27.6)	0,663^a^
Anemia (< 11 g/dL)	16 (26.67)	79 (28.31)	0,796^b^
Glucosa en ayunas (mg/dL)	60 (57–61.5)	80 (73–86)	< 0,001^a^

PTG-75g: prueba de tolerancia oral a la glucosa con 75 gramos; IMC: índice de masa corporal.

a Variables con distribución no paramétrica presentadas como mediana (intervalo intercuartil) y comparadas con la prueba U de Mann-Whitney.

b Frecuencias presentadas como n (%) y comparadas con las pruebas *X*^2^ de Pearson o exacta de Fisher.

### Resultados Neonatales

Los neonatos del grupo GA < percentil 10 se caracterizaron por pesos al nacer y percentiles IG21 más bajos, así como por tener mayores frecuencias de PN < p3, PN < p10 y pesos < 2500 gramos en nacimientos de término (8,6% y 2,3%, p = 0,034). No hubo diferencias entre los grupos con respecto a la edad gestacional al nacer, género neonatal, escala Apgar a los 5 minutos ni en peso < 2500 gramos al incluir nacimientos pretérmino (Tabla 2).

**Tabla 2:** Características neonatales según la glucemia en ayunas en la PTG-75g.

Característica	Glucemia en ayunas		p
	< percentil 10 (n = 60)	≥ percentil 10 (n = 279)	
Edad al nacer (semanas)	39 (38.4–39.6)	39(38.1–40)	0,356^a^
< 34	1 (1.67)	2 (0.72)	0,443^b^
< 37	2 (3.33)	22 (7.88)	0,275^b^
> 41	2 (3.33)	5 (1.79)	0,359^b^
Peso al nacer (gramos)	2947.28 (2833.82–3060.75)	3138.26 (3091.9–3184.7)	0,001^c^
< 2.500	6 (10)	13 (4.66)	0,103^b^
> 4.000	0	3 (1.07)	1^b^
Percentil del peso al nacer IG21	29.32 (9.93–54.61)	49.24 (28.5–70.1)	< 0,001^a^
< 3	5 (8.33)	5 (1.79)	0,006^b^
< 10	15 (25)	19 (6.81)	< 0,001^b^
> 90	2 (3.33)	15 (5.38)	0,511^b^
Género del neonato			
Femenino	24 (40)	145 (52)	0,092^b^
Masculino	36 (60)	134 (48)	0,092^b^
Apgar < 7 a los 5 minutos	5(8.33)	16 (5.7)	0,449^b^

PTG-75g: prueba de tolerancia oral a la glucosa con 75 gramos; IG21: Intergrowth-21st Project.

a Variables con distribución no paramétrica presentadas como mediana (intervalo intercuartil) y comparadas con la prueba U de Mann-Whitney.

b Frecuencias presentadas como n (%) y comparadas con las pruebas *X*^2^ de Pearson o exacta de Fisher.

c Variables con distribución paramétrica presentadas como media (IC 95%) y comparadas con la prueba t de Student.

La correlación entre GA y peso al nacer fue significativa en el grupo GA < p10 (Coeficiente de correlación de Spearman = 0,274, IC 95% 0,22–0,49, p = 0,034).

### Predicción de Peso Bajo al Nacer

El análisis de regresión logística multivariante se utilizó para establecer la asociación entre GA < percentil 10 con PN < p3, PN < p10, peso < 2500 gramos y características maternas mientras se controlaron variables potencialmente confusoras como: edad materna, IMC durante la PTG-75g, ganancia de peso gestacional, paridad, anemia y género neonatal.

De las características maternas, la única significativamente asociada con PN < p10 fue la nuliparidad con un OR 2,28, IC 95% 1.07–4.83, p = 0,032. Esta variable fue utilizada para construir el modelo basal para predecir BPN con R^2^ de Nagelkerke de 2,9% y ABC de 0,60, IC 95% 0.55–0.65. La GA < percentil 10 estuvo independientemente asociada con riesgos incrementados de PN < p3 (OR 4,98, IC 95% 1.4–17.8, p = 0,018), PN < p10 (OR 4,56, IC 95% 2.16–9.63, p < 0,001) y pesos < 2500 gramos (OR 3,95, IC 95% 1.16–13.41, p = 0,04), pero esta última únicamente en embarazos de término. El modelo de predicción basado en la GA < percentil 10 tuvo R^2^ de Nagelkerke de un 8,8%. Al añadir al modelo basal la GA < percentil 10 se mostró un R^2^ de Nagelkerke de un 12,2%, ABC 0,72, IC 95% 0.67–0.77 mejorando significativamente el modelo previo (DeLong: p = 0,005). En la Tabla 3 se detallan las características de los modelos de regresión logística creados y las comparaciones entre ellos. La Figura 2 muestra las ABC de los modelos creados.

**Tabla 3:** Modelos construidos para la predicción de bajo peso al nacer establecido mediante IG21.

Modelo	OR	IC 95%	p	ABC	IC 95%	Sen (%)	Esp (%)	VPP (%)	VPN (%)
Características maternas: Nuliparidad	2,28	1,07–4,83	0,032	0,60	0,55–0,65	67,61	52,15	13,63	93,52
GA < percentil 10	4,56	2,16–9,63	< 0,001	0,65	0,59–0,70	44,13	85,29	25	93,21
Nuliparidad + GA < percentil 10	2,52 4,91	1,16–5,49 2,29–10,52	0,022 < 0,001	0,72	0,67–0,77^a^	23,53	93,44	28,67	91,61

1 IG21: Intergrowth-21st; ABC: área bajo la curva; GA: glucemia en ayunas.

a Comparación de ABC con modelo previo, DeLong: p = 0,005.

**Figura 2:** Desempeño de los modelos predictivos de peso al nacer < percentil 10 según Intergrowth-21st basados en la glucemia en ayunas de la PTG-75g.

## DISCUSIÓN

Estos resultados presentan que, al excluir los factores de riesgo y controlar variables confusoras con potencial efecto negativo sobre el peso fetal, la GA < percentil 10 durante la PTG-75g predice independientemente el BPN. La única característica materna, además de la GA < percentil 10, con influencia sobre el peso al nacer fue la nuliparidad, y el resultado de combinar ambas variables fue el incremento de su capacidad para predecir BPN.

Aunque múltiples factores de riesgo han sido citados como posibles causas de RCIU, muchos de estos fetos permanecen sin detectarse por ultrasonografía hasta el nacimiento y una gran cantidad de las causas metabólicas no han sido establecidas^10^^,^^23^.

Algunos fetos con peso menor que el percentil 10 para la edad gestacional de causa no explicable tienen hipoglucemia y en estos embarazos las madres tienen una sensibilidad a la insulina 22% mayor con niveles plasmáticos de glucosa, insulina y lactógeno placentario más bajos después de la prueba de tolerancia a la glucosa comparadas con aquellas con fetos con peso normal^7^^,^^25^. Es probable que la principal causa de hipoglucemia en estos fetos sea un aporte disminuido de glucosa proveniente de la madre y/o un aumento del consumo placentario de glucosa pero no una disminución en la densidad placentaria de transportadores de glucosa ni diminución de su capacidad de transporte a través del sincitiotrofoblasto^25^^,^^26^.

La identificación de la causa del BPN es crítica para establecer estrategias de prevención y tratamiento. No siempre es posible atribuirlo a factores de riesgo bien conocidos como aneuploidías, defectos estructurales, insuficiencia placentaria o infecciones^27^. Lo anterior fue comprobado en el presente estudio al excluir mujeres con dichas características y con la observación de que solamente un 8,8% de la varianza del PN < p10 fue explicada por la GA <percentil 10, por lo tanto, no podría establecerse una relación causal.

Los datos presentados son similares a investigaciones previas que encontraron relación entre niveles bajos de glucosa sérica durante el tamizaje de DG con BPN^3^^–^^6^. Estudios como el de Naik et al. utilizaron puntos de corte de glucosa sérica más bajos (≤ 50 mg/dL) para definir hipoglucemia y no han encontrado asociación con bajo peso al nacer^28^, pero a diferencia de la presente investigación incluyeron embarazos mayores de 28 semanas con diagnóstico de DG en tratamiento con insulina, no diferenciaron otros factores de riesgo y utilizaron dispositivos de monitorización continua de glucosa que registraron > 90% de los episodios de hipoglucemia entre las 23:00 y las 06:00 horas sin relación con la PTG-75g. El uso de ese punto de corte en nuestra población no tendría aplicabilidad clínica, pues la incidencia de GA ≤50 mg/dL habría sido de un 0,86% con ABC para predecir BPN de 0.51, IC 95% 0.46–0.57.

De manera similar a otros reportes, tampoco encontramos relación con nacimientos pretérmino, Apgar bajo ni morbilidad materna^5^^,^^10^^,^^27^. Por otra parte, estos resultados contrastan con los de Calfee et al.^29^, quienes no encontraron diferencia en la incidencia de RCIU entre mujeres con niveles bajos de glucosa y aquellas con glucosa normal. En ese estudio, el grupo de mujeres con glucosa baja tenían menor edad y peso, e incluía un mayor número de adolescentes y nulíparas que el grupo control. Estas diferencias con nuestra investigación podrían explicarse al considerar lo siguiente: no hubo diversidad étnica en la población estudiada, las características basales de los grupos estudiados fueron homogéneas y a pesar de que buscamos controlar las categorías del IMC como factor de riesgo para BPN, el 59% de las pacientes incluidas tenían sobrepeso u obesidad, lo cual pudo disminuir la sensibilidad a la insulina materna y modificar el crecimiento fetal.

En este análisis, la edad materna no fue un factor asociado con BPN, contrario al estudio de Pugh et al. que se describió que las mujeres con hipoglucemia eran más jóvenes y con IMC pregestacional más bajo^30^. Esta diferencia puede basarse en que esas mujeres tenían, además, enfermedades previas al embarazo que podían modificar el peso al nacimiento, ítem que en la presente investigación fue excluido con la intención de evitar el sesgo de confusión.

Nuestra investigación tiene varias fortalezas, hasta el momento sabemos que es el primer estudio nacional que reportó el percentil 10 de GA durante la PTG-75g en mujeres embarazadas, así como la asociación entre la GA menor a ese punto de corte con el peso neonatal clasificado con IG21. Además, fue el diseño prospectivo en el que a diferencia de los estudios previos (en su mayoría retrospectivos)^3^^,^^5^^,^^6^^,^^16^^,^^27^, se excluyeron y controlaron estrictamente la mayoría de los factores de riesgo relacionados con fetos pequeños para la edad gestacional y la RCIU.

Algunas limitaciones deben considerarse: primera, un potencial sesgo de selección porque se realizó el estudio en un único centro de referencia de tercer nivel con alta prevalencia de DG y factores de riesgo que podrían afectar el crecimiento fetal. Segunda, aunque se trató de excluir y controlar características consideradas factores de riesgo para RCIU y BPN, podría haber factores maternos, placentarios o fetales aún desconocidos con el potencial de modificar el peso al nacer. Tercera, este estudio incluyó únicamente a mujeres mexicanas, por lo tanto, la validez externa podría estar limitada por la homogeneidad de la cohorte.

Finalmente, potenciales variables de confusión que podrían modificar el peso al nacer como nivel socioeconómico, IMC pregestacional, ingesta calórica durante la gestación y nivel de lípidos séricos no fueron obtenidas.

## CONCLUSIÓN

En mujeres embarazadas sin factores de riesgo que pudieran modificar el peso fetal, el modelo predictivo creado combinando GA < p10 durante la PTG-75g con nuliparidad estuvo asociado significativamente con el incremento en el riesgo de BPN. Aunque la capacidad discriminatoria del modelo propuesto fue moderada, podría representar una herramienta clínica útil para identificar mujeres con riesgo alto de tener un neonato con bajo peso al nacer, además de proporcionar una oportunidad para posibles acciones profilácticas, así como para diseñar modelos predictivos que involucren parámetros ultrasonográficos fetales con la finalidad de disminuir la tasa de morbimortalidad infantil relacionada con BPN.
